# Characteristics of Food Protein-Derived Antidiabetic Bioactive Peptides: A Literature Update

**DOI:** 10.3390/ijms22179508

**Published:** 2021-09-01

**Authors:** Nhung Thi Phuong Nong, Jue-Liang Hsu

**Affiliations:** 1 Department of Tropical Agriculture and International Cooperation, National Pingtung University of Science and Technology, Pingtung 91201, Taiwan; npnhung91@gmail.com; 2 Department of Basic Science, Thainguyen University of Agriculture and Forestry, Quyetthang Ward, Thai Nguyen 250000, Vietnam; 3 Department of Biological Science and Technology, National Pingtung University of Science and Technology, Pingtung 91201, Taiwan; 4 International Master’s Degree Program in Food Science, National Pingtung University of Science and Technology, Pingtung 91201, Taiwan; 5 Research Center for Animal Biologics, National Pingtung University of Science and Technology, Pingtung 91201, Taiwan

**Keywords:** DPP-IV inhibitors, PTP-1B inhibitors, α-glucosidase inhibitors, type 2 anti-diabetes, bioactive peptides, bioassay-guided methods

## Abstract

Diabetes, a glucose metabolic disorder, is considered one of the biggest challenges associated with a complex complication of health crises in the modern lifestyle. Inhibition or reduction of the dipeptidyl peptidase IV (DPP-IV), alpha-glucosidase, and protein-tyrosine phosphatase 1B (PTP-1B) enzyme activities or expressions are notably considered as the promising therapeutic strategies for the management of type 2 diabetes (T2D). Various food protein-derived antidiabetic bioactive peptides have been isolated and verified. This review provides an overview of the DPP-IV, PTP-1B, and α-glucosidase inhibitors, and updates on the methods for the discovery of DPP-IV inhibitory peptides released from food-protein hydrolysate. The finding of novel bioactive peptides involves studies about the strategy of separation fractionation, the identification of peptide sequences, and the evaluation of peptide characteristics in vitro, in silico, in situ, and in vivo. The potential of bioactive peptides suggests useful applications in the prevention and management of diabetes. Furthermore, evidence of clinical studies is necessary for the validation of these peptides’ efficiencies before commercial applications.

## 1. Introduction

Nowadays, food components are of great concern because of their high nutritional content and role in the prevention and management of diabetes. Due to the rapid development of potential bioactive compounds from natural sources, food proteins or food-derived peptides have been recognized to play an essential role in regulating blood glucose levels, which is one of the characteristics of diabetes [1,2,3,4]. Numerous studies of potential peptides are being carried out in preclinical trials [5,6]. During the period of 2015–2019, the United States Food and Drug Administration (FDA) approved a total of 208 new drugs, of which peptides or peptide-containing molecules accounted for approximately 7%, suggesting the importance of therapeutic peptides in the pharmaceutical industry [6]. As mentioned in literature, bioactive peptides can only be released from protein parents under specific conditions such as enzyme digestion, chemical and physiological mechanisms, and microbial fermentation [7,8,9]. Generally, bioactive peptides range between two to twenty residues in length and play a role in vital and positive health benefits on the human body [10,11,12]. Regularly, the bioactivity characteristics of peptides have been correlated to several factors such as hydrophobicity, the location of special amino acids in sequences, and interaction with the active site of target enzymes [2,7,8,13,14].

Diabetes mellitus is a chronic metabolic disorder resulting from insufficient active insulin or abnormal response to insulin, and leading to high levels of blood glucose (hyperglycemia) [7,15]. Insulin hormone, induced from beta cells of the pancreas, is responsible for the regulation of the circulating blood glucose derived from carbohydrate hydrolysis in foods [16]. Type 1 diabetes is caused by the inability of the pancreas to produce insulin. Type 2 diabetes (T2D), estimated as the cause for 90–95% of all diabetes, occurs when insulin receptors cannot respond to insulin, thereby causing insufficient insulin expression or insulin resistance [16,17,18]. As a long-term complication, T2D can provide favorable conditions for the development of other metabolic diseases. For instance, hypertension and diabetes have a common metabolic pathway and therefore it is highly possible that both diseases can occur in the same individual [19]. In addition, diabetic patients have been recognized as high risks for complications such as cardiovascular disorders or the recent COVID-19 disease, leading to increased severity, difficulty in curing the disease, and even mortality [17,20,21,22,23].

Numerous therapies have been established for the prevention and the development constrain of diabetes through which the main principle is the management of postprandial hyperglycemia. One widely used therapeutic approach for the control of T2D is through the reduction of glucose levels by inhibition of the dipeptidyl peptidase IV (DPP-IV) enzyme, consequently maintaining the concentration of glucagon-like peptide-1 (GLP-1) and glucose-dependent insulinotropic polypeptide (GIP), two insulin expression inducers [18,24,25]. Another therapeutic approach is the use of α-glucosidase inhibitors to inhibit carbohydrate cleavages in the small intestine [26,27,28]. Lastly, maintaining the insulin activity status using protein-tyrosine phosphatase 1B (PTP-1B) inhibitors is another approach mentioned [16,29]. Currently, food-derived antidiabetic peptides with lesser side effects than commercial drugs have been isolated from various dietary proteins [15,16,20]. Different food-protein hydrolysates from milk, meat products, plants, and marine organisms are high recommendations as excellent sources of bioactive peptides [2,3,30,31,32,33,34,35,36,37,38].

This review is aimed at providing a comprehensive understanding of the association between active peptides and antidiabetic effects through a two-strategy approach. The first part provides an overview of the antidiabetic inhibitors released from food proteins. The inhibitory characteristics of DPP-IV, PTP-1B, and α-glucosidase enzyme focuses on their mechanisms, efficiency, and safety. The second part lays out the status of updated knowledge on the screening methods and determines the bioactivity feature of novel DPP-IV inhibitory peptides. This approach is elucidated via fractionation and purification methods, peptide identification approaches, in vitro assay, the sequence—activity correlation, modes of action, stability towards DPP-IV or gastrointestinal digestive enzymes, and quantification of bioactive peptides. Moreover, molecular docking analysis is also used to investigate the interaction relationship between potent inhibitory peptides and the DPP-IV. Furthermore, the cell model with advantages and disadvantages to studying food-derived peptides is introduced. Subsequently, in vivo research evidence from animals and humans is supplemented to support the potential of bioactive peptides in the prevention and management of diabetes.

## 2. Dietary Proteins as Precursors of Anti-Diabetes Peptides

### 2.1. Dipeptidyl Peptidase—IV Inhibitors

**Figure 1:**
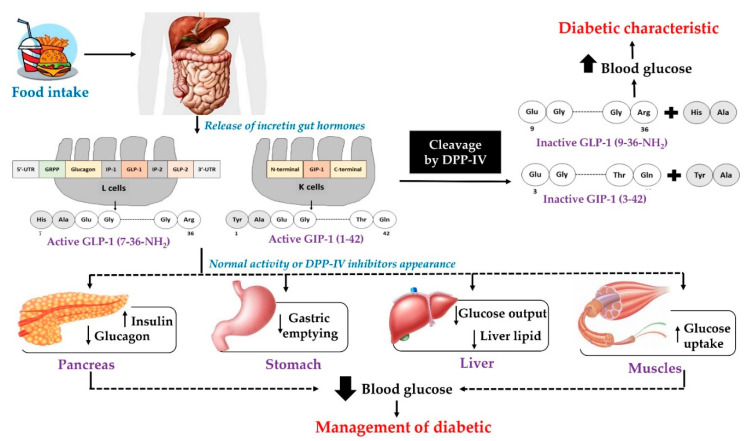
Schematic representation of the mechanism for the glucose-lowering action of DPP-IV inhibitors. GLP-1 and GIP are substrates for the DPP-IV enzyme that hydrolyzes them into shorter and inactive molecules. Note: DPP-IV, dipeptidyl peptidase IV; GIP, glucose-dependent insulinotropic polypeptide; and GLP-1, glucagon-like peptide-1.

Dipeptidyl-peptidase IV (DPP-IV; EC 3.4.14.5) is known as the lymphocyte cell surface marker CD26 or the adenosine deaminase (ADA)-binding protein [15,24]. This enzyme is a 110 kDa plasma membrane glycoprotein that belongs to the prolyl oligopeptidase family. In addition, the DPP-IV enzyme acts as a serine protease that preferentially cleaves N-terminal dipeptides from oligopeptides, particularly if alanine or proline is located in the second position [39]. Since its first description in rat liver, DPP-IV activity has been detected in various mammals, microorganisms, and plants. In humans, the enzyme is expressed in almost all organs and tissues, especially in the intestine, bone marrow, pancreas, spleen, and kidney [40]. Interestingly, the DPP-IV sequence has been regarded as highly conserved among species [41,42]. Additionally, the DPP-IV takes part in various biological processes such as enzymatic incretin inactivation; prevention of cancer growth; immune and endocrine activity; and cell adhesion [40,43]. This functional enzyme is mainly responsible for degrading incretin hormones, glucagon-like peptide-1 (GLP-1) and glucose-dependent insulinotropic polypeptide (GIP) [18,24,25]. GLP-1 is induced by translational processing of proglucagon with two active forms, namely GLP-1 (7-36) amide and glycine-extended GLP-1 (7-37), while GIP, a 42 amino acid protein (GIP 1-42), is released from the proGIP precursor [17,44]. Released after meal ingestion, both hormones can promote insulin secretion for up to approximately 60% of the total insulin from pancreatic β cells, thereby regulating postprandial blood glucose (Figure 1) [45,46]. In the presence of DPP-IV, it has been estimated that the percentage of inactivated GLP-1 secreted during a meal is more than 95% [47]. Insufficiency of the insulin activation level principally causes hyperglycemia, commonly determined in T2D patients. Increasing insulin concentration may be possible via longer half-life incretin hormones, hence resulting in decreased glucose levels in the blood. Therefore, the development of potential DPP-IV inhibitors is considered as a therapeutic strategy for glucose-lowering treatment in the management of diabetes.

To date, the most widely available DPP-IV inhibitors with FDA approved labels as diabetes drugs include sitagliptin, linagliptin, vildagliptin, saxagliptin, and alogliptin [20]. In general, most synthetic DPP-IV inhibitors are well tolerated by consumers, although the FDA also warned about their side effects. As in clinical trials, the risk of joint pain was of concern with four of the inhibitors, except vildagliptin. Moreover, the use of saxagliptin and alogliptin is associated with increases in the risk of heart failure. In addition, adverse cardiovascular problems have also been accompanied by sitagliptin. Peptides derived from edible proteins are generally regarded as safe and easy-to-be-absorbed nutrients of amino acid supplements. In other words, the discovery of food-derived DPP-IV inhibitory peptides enhances its contribution to the more efficient and safe as therapeutics of T2D. Many DPP-IV inhibitory peptides have been reported in common dietary hydrolysate proteins from milk [14,48,49], plants [50,51,52,53], and marine organism proteins [54,55,56]. The generation, isolation, identification, in vitro/in silico/in vivo evaluation, and mechanism study of DPP-IV inhibitory peptides will be discussed specifically in Section 3.

### 2.2. PTP-1B Inhibitors

Protein-tyrosine phosphatase 1B (PTP-1B), also called protein tyrosine phosphatase non-receptor type 1, is a member of the protein tyrosine phosphatases (PTPs; EC 3.1.3.48) family that plays a role in controlling T2D and obesity [16,29]. In the human body, the enzyme is expressed in various organs such as adipose tissue, liver, muscle, and brain [57]. This enzyme encoded by the PTPN1 gene, which is known for a catalytic feature to hydrolyze phosphate groups from tyrosine-phosphorylated proteins. Specifically, the PTP-1B cleaves the phosphate group from crucial tyrosine residues on the activated insulin receptor, causing the inactivation of insulin. Therefore, the inhibition of the PTP-1B expression can ameliorate insulin resistance, leading to an increase in glucose uptake in an insulin-responsive cell, which is considered an important key for a therapeutic target for the treatment of diabetic patients. Synthetic PTP-1B inhibitors ertiprotafib, ISIS-113715, and trodusquemine failed in clinical trials or presented undesirable side effects; however, a glitazone with the thiazolidine-2,4-dione (TZD) structural feature is considered as a promising inhibitor [16].

In recent years, natural PTP-1B inhibitors have also been reported in a variety of dietary substrates such as blueberry, marine sponges, *Pueraria lobata* root, and Chinese raspberry [58,59,60,61,62]. In actuality, the majority of published studies have focused on flavonoids, phenolics, terpenoids, steroids, and alkaloids, but no research has been published on peptides [60,61,63,64,65,66,67]. Flavonoids isolated from the medicinal plant *Morus alba* L. (Moraceae) with four Diels–Alder type adducts (morusalbins A−D) are known for dual functionality in the inhibition of the PTP-1B enzyme (IC_50_ value of 1.90−9.67 μM) and α-glucosidase (IC_50_ value of 2.29−5.91 μM) [68]. Moreover, the role of PTP-1B in the negative regulation of insulin sensitivity and metabolism was also determined in specific tissue models and animal models. Three components including mulberrofuran G, albanol B, and kuwanon G released from *Morus alba* L. root bark have been reported to significantly enhance glucose uptake and reduce the concentration of the PTP-1B enzyme in a dose-dependent manner in insulin-resistant HepG2 cells. In addition, the α-glucosidase inhibition effects were also recognized in *Morus alba* L. root bark with IC_50_ values of 1.67–2.35 μM [67]. In another medicinal plant study, the six derivatives of moronic acid (1−6), collected from *Phoradendron reichenbachianum*, were examined using in vitro, in silico, and in vivo experiments for antidiabetic effects. Among them, derivative 6 performed the best PTP-1B inhibition activity with the IC_50_ value of 7.5 ± 0.1 μM and effectively reduced the presence of plasma glucose in vivo in the non-insulin diabetic rat model [69]. Additionally, *Pueraria lobata* root-derived triterpenoids lupeol and lupenone possessed respective IC_50_ values of 38.89 ± 0.17 μM and 15.11 ± 1.23 μM for the PTP-1B inhibition activity [61]. Furthermore, anthocyanins from blueberry [59], furanosesterterpenes from two Indonesian marine sponges, namely *Ircinia* and *Spongia* spp. [58], and cladosporamide A derived from an Indonesian marine sponge-derived *Cladosporium* sp. [60] have been recognized to possess PTP-1B inhibitory effects.

### 2.3. α-Glucosidase Inhibitory Peptides

**Figure 2:**
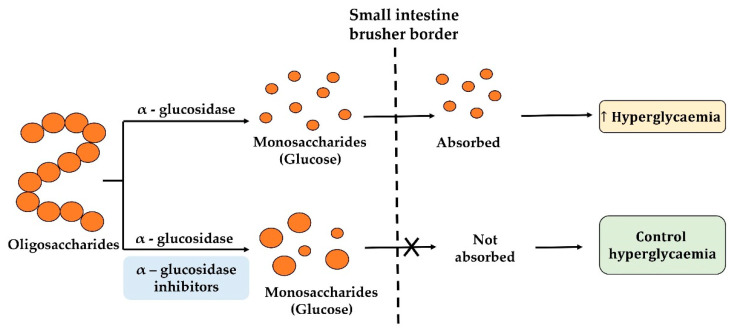
Mechanism of action of alpha-glucosidase inhibitors.

The α-glucosidase (EC 3.2.1.20), located in the small intestine, is the main catalytic enzyme responsible for the cleavage of carbohydrates down to free glucose molecules, resulting in a rise in blood glucose concentration after meals [26,27,28]. The enzyme’s feature is the preferential hydrolysis of non-reducing α-(1 → 4), α-(1 → 3) and α-(1 → 2) linked D-glucose residues [34]. Therefore, the use of α-glucosidase inhibitors is considered an effective strategy to maintain lower serum glucose levels in the management of T2D (Figure 2). The inhibitors targeting this enzyme act to retard carbohydrate metabolism, delay the time of glucose absorption, and ultimately result in the decrease of postprandial hyperglycemia. Traditionally, several α-glucosidase inhibitors such as acarbose, voglibose, miglitol, and emiglitate have been widely used to manage the level of blood glucose in diabetic patients [15]. However, adverse side effects such as flatulence, diarrhea, vomiting, and abdominal cramping have also been reported. Therefore, numerous peptides have been identified and isolated from dietary proteins displaying α-glucosidase inhibition activity.

### 2.4. Food Protein-Derived α-glucosidase Inhibition Peptides

To date, various protease-digested plant hydrolysates from beans, *Ocimum tenuriflorum* seeds, hemp seed protein, walnut, soybean, rice, and dark tea have been indicated as the main contributors to inhibit α-glucosidase [27,31,70,71,72,73,74,75]. Small peptides generally possess more potential α-glucosidase inhibitors as compared to large peptides. Germinated soybean-derived peptides with a molecular weight of <5 kDa showed the best inhibition activity compared with larger sized peptides (5–10 kDa and >10 kDa) [72]. Comparably, higher α-glucosidase inhibitory activities were recognized in peptides with a small molecular weight (<3 kDa) than the crude digests of rice bran proteins [73].

Two novel peptides (WH and WS) released from almond oil manufacture residue revealed potent α-glucosidase inhibitory activity. The peptide WH is stable in simulated gastrointestinal digestion, with the ability to maintain the IC_50_ value of the α-glucosidase inhibitory effect (17.03 ± 0.05 μmol/L), while WS significantly increased the IC_50_ after simulated digestion (24.71 ± 0.02 μmol/L to 44.63 ± 0.03 μmol/L) [37]. Ibrahim et al. reported that peptide SEPA, selected from 844 peptides (BIOPEP database) using molecular docking simulation, can exhibit an α-glucosidase inhibitory activity with an IC_50_ value of 0.79 mM, which was several-folds lower than acarbose (IC_50_ = 1.72 mM) [76]. Moreover, milk and meat products with high protein contents were also regarded as peptide sources with multifunctional bioactivity [34,77,78]. Eight peptides isolated from Spanish dry-cured ham protein were reported to possess multiple functionalities such as antioxidant effects, ACE inhibition, DPP-IV inhibition, and low IC_50_ values (5.58 to 26 mM) for α-glucosidase inhibitory activity [34]. Interestingly, peptides derived from species of edible insects also displayed ACE, α-glucosidase, and lipase inhibitory activities [13]. Among four peptides released from silkworm, pentapeptide SQSPA performed the highest inhibition activity against α-glucosidase with IC_50_ values of 20 µmol/L [28] (Table A1).

## 3. Common Acquisition Procedures for DPP-IV Inhibitory Peptides

### 3.1. The Strategy of the DPP-IV Inhibitory Peptide Discovery

**Figure 3:**
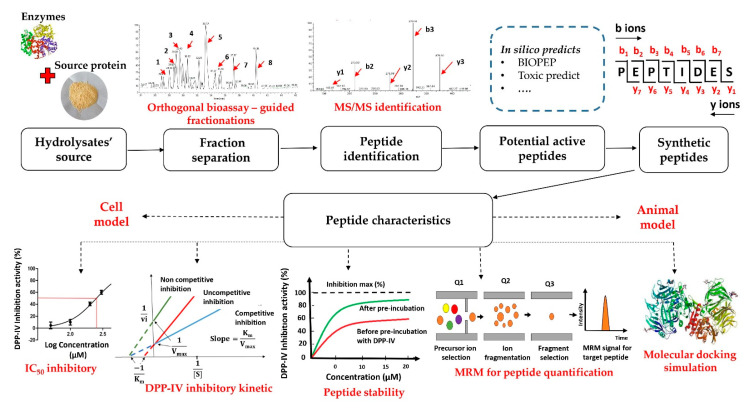
Workflow for discovering DPP-IV inhibitory peptides from food-derived proteins.

Towards incorporating more innovative functional foods or drugs into the pharmaceutical system for diabetes treatment, novel DPP-IV inhibitors should be completely evaluated for their effectiveness and safety. Three major assessment systems regularly carried out in the above mission are in vitro assay of inhibition, in situ inhibition in cell culture, and in vivo/ex vivo estimation in small animals. In this review, more emphasis is placed on the use of in vitro assay for DPP-IV inhibition identification. The methods currently employed to discover DPP-IV inhibitory peptides are illustrated in Figure 3. The strategy of active peptide determination is executed as follows: (i) generation of peptides; (ii) bioassay-guided fractionation (separation or fractionation + in vitro DPP-IV inhibitory assay); (iii) peptide identification using tandem mass spectrometry (MS/MS) coupled with database-assisted identification or de novo sequencing; and (iv) characterization of DPP-IV inhibition properties using purified peptides. Each stage of the workflow is detailed below.

### 3.2. Enzymatic Hydrolysates of Food Proteins

**Figure 4:**
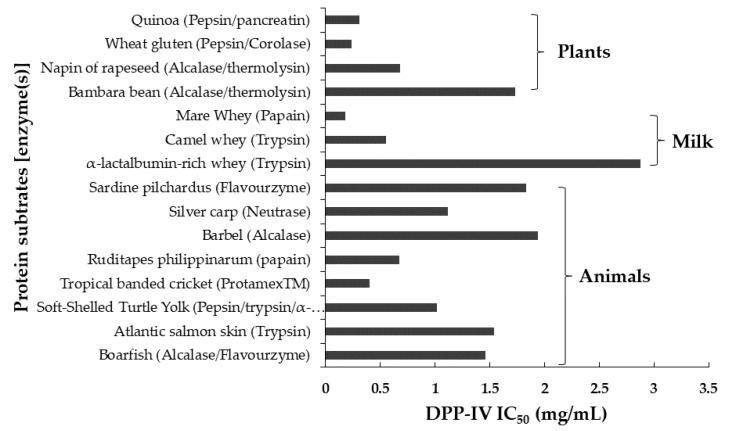
Food protein-derived hydrolysates displaying in vitro dipeptidyl peptidase (DPP-IV) half maximal inhibitory concentration (IC_50_) values (2016–2020).

The dietary proteins with highly diverse sequences used as precursors to generate protein hydrolysates and peptides with DPP-IV inhibitory activities have drastically increased over the years. To date, various food commodities such as milk [4,8,35,49,79], fish [36,54], egg [1], or special ones such as quinoa [53], rapeseed [80], and tropical banded cricket [9] are well-known as DPP-IV inhibitor sources. The IC_50_ values of various DPP-IV inhibitory hydrolysates collected from the data of scientific reports in the last five years (2016–2020) are summarized in Figure 4 (see original data in Table A2). Nowadays, milk protein hydrolysates released from camel, bovine, and mare are generally considered as potential DPP-IV inhibitory sources [4,8,79]. However, other sources such as marine organisms (Boarfish) [36], plant (Brewers’ spent grain and rapeseed) [52,80], and soft-shelled turtle eggs [2] have also attracted consideration as promising DPP-IV inhibitory peptides because of their high proline residue content, an amino acid proven to play a role in the enhancement of DPP-IV inhibition activity. The hydrolysates from tropical banded cricket [9], wheat gluten [81], mare whey [79], and quinoa [53] have been demonstrated with notable DPP-IV IC_50_ indexes lower than 0.5 mg/mL (Figure 4).

In the literature, obtaining bioactive peptides from food proteins is carried out most preferentially by enzymatic hydrolysis, secondarily via microbial fermentation [82,83,84,85], and to a lesser extent with chemical agents [86]. The advantage of the first method is product predictability, reduction of reaction time, and simple application on a large scale. Two independent studies on the discovery of angiotensin-converting enzyme (ACE)-inhibitory peptides derived from milk proteins using different hydrolysis protein methods have been reported. ACE inhibitory peptides were generated by milk fermentation with the *Lactobacillus casei* strain for up to 42 h [87], while bioactive peptides from camel milk protein hydrolysates using papain and alcalase took around 9 h [88]. The main attributes in using microbial fermentation are selecting suitable strains for specific proteins, setting a temperature fitting for bacterial growth, and controlling the fermentation time. The performance of enzymatic hydrolysis to produce bioactive peptides will be discussed in this section. Generation of DPP-IV inhibitory peptides from food proteins can be achieved using a single protease or multiple enzymes obtained from plants (e.g., papain) [49,79,89,90], animals (e.g., pepsin, trypsin, and Corolase PP) [2,3,35,81], or microorganisms (e.g., alcalase, protamex, and flavourzyme) [9,91,92,93]. Each protease requires the provision of specific conditions (e.g., pH and temperature, enzyme/substrate ratio, and hydrolysis time) to optimize hydrolysis efficiency or the DPP-IV inhibitory effect of hydrolysate. Alternatively, the water-soluble extract from natto is an exceptional case whereby hydrolysates were created during food processing without any exogenous enzyme digestion, but the DPP-IV IC_50_ value (5.35 mg/mL) seemed still high [94]. Additionally, the degree of hydrolysis, a main parameter to define the hydrolysis efficiency, is determined as the proportion of cleaved peptide bonds in a protein hydrolysate [95]. After enzymatic digestion, a high value for the degree of hydrolysis indicates better intestinal absorption and more accessibility to the active sites of DPP-IV. Therefore, DPP-IV inhibitory hydrolysates with the lowest IC_50_ values could be selected and further isolated for bioactive peptide identification.

### 3.3. Bioassay-Guided Fractionation Methods

Generally, inhibitors with smaller sizes perform better DPP-IV inhibitory activity than bigger-sized ones. Peptides with different molecular sizes are separated using molecular weight cut-off (MWCO) membranes based on pore size. In the studies of Zhang et al., the MWCO membranes were utilized to separate Silver carp and Bighead carp protein hydrolysates into <3 kDa, 3–5 kDa, 5–10 kDa, and >10 kDa peptide fractions [90,96] in which the highest DPP-IV inhibition effect was performed by <3 kDa fractions. Similarly, soft-shelled turtle yolk hydrolysates and Natto protein with a molecular weight of <3 kDa also indicated the inhibition enhancement towards DPP-IV [2,94]. In agreement with this trend, fractions smaller than one kDa showed the best DPP-IV inhibition activity as compared to the other fractions (3 kDa, 5 kDa, and 10 kDa) found in the hydrolysate of rapeseed [80], tropical banded cricket [9], and wheat gluten [81].

The bioactive peptides derived from protein hydrolysates usually coexist in a mixture carrying inferior peptides. Therefore, the enhancement of the peptide separating efficiency, leading to reduced researching time, financial hardship, and human resources of laboratories, is a foremost mission in the strategy for discovering novel potential DPP-IV inhibitory peptides. The main physicochemical properties of peptides are molecular weight, charge, hydrophobicity, and hydrophilicity. The preferential bioassay-guided fractionation methods are reversed-phase chromatography, size exclusion chromatography (SEC), and strong cation exchange (SCX) chromatography [2,41,81,90,97]. The reversed-phase chromatography is the most widely used, including reversed-phase high-performance liquid chromatography (RP-HPLC), semi-preparative reversed-phase high-performance liquid chromatography (SP-RP-HPLC), and reversed-phase ultra-high performance liquid chromatography (RP-UPLC). Their separation mechanism mainly depends on the hydrophobic properties of amino acids. Unlike the reversed-phase technique, peptides are isolated using size exclusion chromatography and strong cation exchange chromatography based on their size and cationic components, respectively. Frequently, the combination of two chromatography methods are sufficient to achieve peptide purification. The most active fraction from the first separation method is subsequently exposed to the second kind of fractionation, coupled with in vitro DPP-IV inhibitory assay to isolate the most active fraction which contains the most potent active peptide. For example, reversed-phase ultra-high performance liquid chromatography (UPLC) and gel permeation high-performance liquid chromatography (GP-HPLC) were used to separate fractions from camel whey protein hydrolysate-releasing peptides that possess potential DPP-IV inhibition activity with an IC_50_ value of 6.6 µM [8]. Gao et al., Gu et al., and Song et al. combined the reversed-phase C18 column of RP-HPLC and gel filtration chromatography in tandem to obtain the fractions with the most potent DPP-IV inhibitory activity [4,38,79]. Moreover, Sila et al. reported the incorporation of three chromatography methods to study barbel protein hydrolysate [83]. This hydrolysate was fractionated by size exclusion chromatography using a Superdex^®^ column. Then, the fraction with the greatest inhibition activity was further fractionated more than two times by RP-HPLC and the peptide sequence of the only recognized peak was identified by MS/MS analysis. However, the identification of the most potent peptide through sequential bioassay-guided fractionation might sometimes omit other potential peptides from the best fraction, especially in the case of complex hydrolysates. The characterization of the possible active peptides that co-existed in the most active fraction would require validation using multiple synthetic peptides, which is a costly, labor-intensive, and time-consuming process. To circumvent that problem, a so-called orthogonal bioassay-guided fractionation has been carried out using two independent fractionations to screen and reduce the number of candidate peptides by selecting the identical peptides simultaneously appearing in both active fractions [98]. For example, Nong et al. used strong cation exchange chromatography and RP-HPLC in parallel to identify the DPP-IV inhibition peptides from soft-shelled turtle yolk hydrolysate [2]. In their study, only five peptides, including VPGLAL, WLQL, LPLF, LVGLPL, and LPSW, were identically identified from the most active fractions of SCX and RP separations.

### 3.4. Peptide Sequence Identification Using Tandem Mass Spectrometry (MS/MS) Analysis Coupled with Database-Assisted Sequence Matching or de Novo Sequencing

Regularly, tandem mass spectrometry (MS/MS) is a suitable tool allowing for the identification and quantification of peptides derived from the most bioactive fractions after bioassay-guided fractionation methods. Mass spectrometry has received high recommendations as a selective and sensitive method for proteomic analysis [99,100,101]. However, insufficient purification of samples containing the interference of endogenous components such as proteins, carbohydrates, salts, and lipid could cause negative impacts on the ionization efficiency and lead to poor sensitivity and reproducibility. To resolve this issue, solid-phase extraction (SPE) is an ideal sample preparation method to eliminate unnecessary components from target peptides via suitable SPE columns and elution solutions [101].

Generally, the MS/MS technique is based on measuring masses of fragment ions of a certain precursor (ion produced during an ionization process) [99,102]. The representation of each fragment is recognized through its mass to charge ratio (*m*/*z*) and all fragments in the MS/MS spectrum can be converted into a peptide sequence using database-assisted sequencing via search engine matching within a protein database or de novo sequencing by detecting the residue’s molecular weight based on the mass difference between neighboring fragments [103,104]. In the database-assisted sequence matching strategy, the peptide with the highest PSM (peptide spectrum match) score is considered as a potential candidate for the peptide in query. The PSM score is measured by the total number of identified peptide fragments (e.g., *b*- and *y*- series ions) matched with the predicted fragments in the protein database; therefore, a higher score represents a higher confidence for an accurate peptide identification [99,105,106]. Mascot and Sequest are two common search engines applied in these approaches [102]. A notable advantage of database searching methods is the ability to correctly distinguish between several residues with the same molecular weight (for example, isoleucine and leucine), leading to the provision of undoubted sequences to peptides. However, for species whose genome has not been decoded, the predicted protein database is incomplete. In that case, the database-assisted peptide sequencing can only be compromised to match the MS/MS data with the protein database derived from a similar or relevant species, which may lead to an unsuccessful identification. Ultimately, de novo sequencing is the method to solve that problem through the direct identification of the peptide sequence based on a tandem mass spectrometry spectrum [106,107]. The sequence of amino acid residue can be manually deduced by determining the residue’s molecular weight based on the mass difference between the neighboring fragments or automatically calculated using some commercially available software. According to previous studies, the PEAKS software is evaluated as the most reliable and accurate for high-quality ESI QqTOF data among de novo sequencing tools [104,108]. For de novo sequencing, the more accurate the measurement of the residue mass, the more reliable the determination of the peptide sequence. Therefore, for peptide de novo sequencing, high-resolution tandem mass spectrometry is preferred.

Unfortunately, de novo sequencing methods can not directly recognize the difference between isoleucine and leucine residue, which may cause a wrong assignment of the peptide sequence. To solve this problem, Armirotti et al. assessed ten synthetic peptides with differences in thevIle/Leu residue position in the primary structure in consecutive low-energy ESI-TRAP MS^n^ experiments [109]. Under the multi-stage fragmentation in an ion trap mass spectrometer, the immonium ions with the same molecular weight to Ile and Leu (86 Da and 113 Da, respectively) residues were further dissociated into two distinct products. Unlike Leu which presented the general fragmentation of both amino acids as 30 Da and 44 Da ions, Ile introduced an additional 69 Da characteristic product. Therefore, the display of Leu and Ile in peptide sequencing could be accurately discriminated. Interestingly, according to the innovation of analysis technology, to date, the distinction of isobaric residue (Xle) as either Ile or Leu can be carried out accurately and rapidly (within 2 days) by applied Nanoflow LC-MS^n^ with the Orbitrap Fusion [110]. This methodology is based on either the generation of a diagnostic 69 Da ion from Ile (also known as the “immonium ion method”) or the formation of corresponding unique w-ions from side-chain losses of z-ions employing MS^3^ (electron capture dissociation (ETD), high energy collision dissociation (HCD)) (called the “unique w-ion method”) for fast Ile/Leu distinction. Upon HCD fragmentation, the production of a peak with 43 Da (loss of isopropyl) or 29 Da (loss ethyl) lesser than the precursor by the z ion is characteristic of Leu and Ile residues, respectively. The “immonium ion method” is preferentially applied with the peptide that contains a single Xle residue and the “unique w-ion method” is more suitable with complicated peptides containing multiple Xle residues in their sequence.

### 3.5. In Silico Prediction of Potential DPP-IV Inhibitory Peptides

Bioinformatics analyses are remarkably regarded as powerful tools used for the anticipation of the potential bioactive peptides derived from foods. The most notable predictions include DPP-IV inhibition ability (BIOPEP) [111,112], toxic action (ToxinPred), the activity of a molecular-based kind on its molecular features (Quantitative Structure-Activity Relationship, QSAR), and the preferred conformation of a molecule interacted with the key residues in the DPP-IV active site (molecular docking, further discussed in Section 3.6.5).

The BIOPEP (BIOPEP-UWM) database of biologically active peptides has been widely used in studying dietary peptides tending to their putative control of chronic diseases [111,112]. For instance, antihypertension activities through the inhibition of the ACE enzyme (angiotensin-converting enzyme) have been determined in peptides derived from the rice bran protein, read agar, and goat milk [113,114,115]. In addition, BIOPEP-UWM provides an abundance of information on peptides as antidiabetic (DPP-IV, α-glucosidase, of α-amylase), antioxidant, HMG-CoA reductase inhibitor activities, etc. [2,72,111,113,116,117]. The activity prediction of a peptide is calculated based on two quantitative parameters, namely the occurrence frequency value (A) and a potential biological activity (B) [111]. The A value, the frequency of bioactive fragments occurrence in a protein sequence, is analyzed to determine the bioactivity of a peptide present in the query sequence. The B value, a potential biological activity of protein fragments, is determined based on the availability of the IC_50_ or EC_50_ value of peptides from the literature. Therefore, peptides with higher A and B values, especially B values, are forecasted to have better DPP-IV inhibition ability.

The availability of ToxinPred (ToxinPred (osdd.net)), an in silico tool, is usefully applied in predicting the toxicity of target peptides/proteins [118]. ToxinPred also allows for anticipating minimum mutations in peptides for adjusting their toxicity (trend enhancing or reducing) and assuming toxic regions in proteins. The main database was built from 1805 toxic peptides with sizes smaller than 35 residues in their sequences.

Quantitative structure–activity relationship (QSAR), an informatics tool, is usefully applied in active molecular research, especially in drug discovery and extensively in bioactive peptide studying [42,119]. This methodology allows for predicting food-derived active peptide properties based on the relationship between their biological activity and chemical structures, basically understanding the same conformation trending to the same bioactivities. Several DPP-IV inhibitions have been investigated with the QSAR approach to clearly understand their structural characteristics. For example, Nongonierma et al. applied the QSAR model to study milk proteins and then found the notable roles of hydrophobic amino acids at the N-terminal side of peptides with DPP-IV inhibitory properties, especially Trp, Ile, Leu, and Phe residues [48,120]. Moreover, Fitzgerald et al. recently published on the utilization of in silico approaches to evaluate the inhibition activities of bioactive milk peptides against ACE, DPP-IV, and oxidants [14]. To date, in silico programs provide valuable information for predicting the biological activity of novel peptides. Consequently, the combination of different bioinformatics tools may enhance the anticipating efficiency of the DPP-IV inhibitory peptides derived from food proteins. However, in vitro and/or in vivo assays to confirm the peptide’s biological activity are unavoidable.

### 3.6. Characteristics of Inhibitory Peptides

#### 3.6.1. Features and Structure–Function Activity of DPP-IV Inhibitory Peptides

Some of the noticeable matters that have been regularly considered in the investigation of novel DPP-IV inhibition peptides are: firstly, to determine the suitable substrates derived from food-protein sources according to their high nutritional values, availability, and affordable price; and secondly to determine the promising efficiency of the peptides’ DPP-IV IC_50_ index. Based on the aforementioned details, more and more innovative research methodologies are applied to enhance the activity of DPP-IV inhibitory peptides from a wide range of food-protein sources (see more in Section 3.3). The rapid development in both the quality and quantity of scientific reports indicate the potential in this field. The number of peptides that possess a DPP-IV IC_50_ value of <100 µM has significantly increased from only three peptides (in 2012) to nearly 100 peptides (until 2020) (Table 1) [121,122]. Moreover, abundant research studies on the DPP-IV inhibitory peptides derived from food proteins were widely reported in the past three years (Table 1). In these studies, a total of 117 DPP-IV inhibitory peptides varying from 2 to 15 amino acids in their sequences were identified from common dietary proteins such as egg [1,2,30,41], milk [8,35,48,79], and fish [3,36]. The IC_50_ values of those identified peptides ranged from 6.6 µM (VPV) to more than 2000 µM; notably, around 30% of the total peptides possessed the IC_50_ value of <100 µM [8,36,52,80,123]. Until now, the tripeptide Ile-Pro-Ile (IPI), also known as diprotin A, has been considered the most potent DPP-IV inhibitor (IC_50_ = ~4 µM) among the currently reported peptides [42]. After IPI, VPV derived from camel milk is the second most potent DPP-IV inhibitory peptide [8]. The information from Table 1 has shown that most of the discovery about food protein-derived novel DPP-IV inhibitory peptides has been focused on in vitro assay and still lost the connection to cell models and/or animal models. The advantages and disadvantages of these experimental models will be further analyzed and explained in Section 3.7 and Section 3.8 of this review. The investigation mostly focuses on the DPP-IV IC_50_ index, however, it is not sufficient evidence to completely understand promising peptides. For further application of DPP-IV inhibitory peptides in clinical studies, both their mechanism of inhibition action and stability, as well as their molecular interaction with ACE receptors should be of concern.

**Figure 5:**
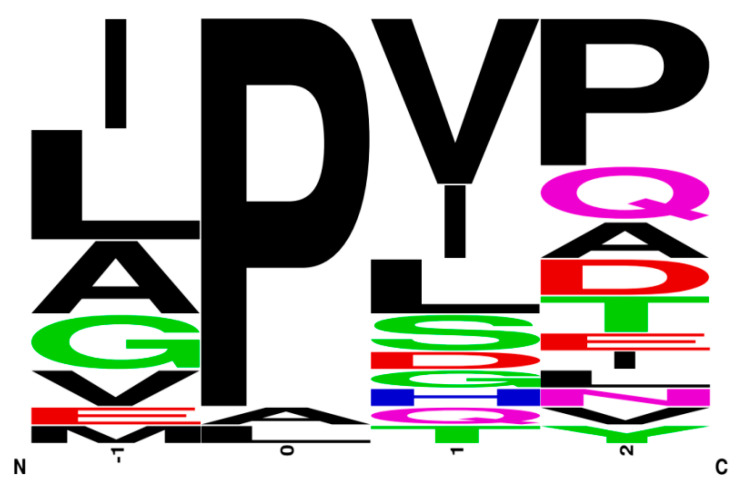
Frequency analysis of amino acid residues contained in highly DPP-IV inhibition peptides (IC_50_ < 100 µM) using WebLogo.

Residue frequencies for the DPP-IV inhibitory peptides that possess an IC_50_ value of <100 µM are summarized using WebLogo (https://weblogo.berkeley.edu/, accessed on 29 August 2021) (Figure 5). WebLogo is the software designed to analyze the sequence conservation from a sequence library. The bigger font size of each letter corresponds to the higher frequency appearance of each amino acid at that position. The amino acids are arranged in order from the most frequent to the least common (from the top to the bottom of each column). Based on different chemical properties, amino acids display various colors such as black presented to hydrophobic residues (A, V, L, I, P, W, F, M), red for acidic (D, E), green for polar (G, S, T, Y, C), pink for polar (Q, N), and basic (K, R, H) amino acids as blue. The hydrophobic residues Ala, Gly, Ile, Leu, Pro, Met, Glu, and Val that frequently appear at the N-terminal position of peptides are represented in Figure 5. The DPP-IV inhibitory activity is significantly affected by the N-terminal of peptides. It suggests that the presentation of Ala, Gly, Ile, Leu, Pro, Met, Glu and Val at the N-terminal of peptides may contribute to high the DPP-IV inhibitory peptides. The interaction of these peptide residues and the hydrophobic pocket of the DPP-IV active site could be a reason to explain their high biological effects. In particular, Pro/Ala (P/A) in the second position of the N-terminal sequence have been associated with DPP-IV inhibitory activity because DPP-IV can determine and preferentially cleave the P/A at this position [42,125]. For example, the top three strongest competitive inhibitors of DPP-IV, including IPI (IC_50_ = ~4 µM), VPV (IC_50_ = 6.6 µM), and VPL (IC_50_ = 15.8 µM), all possess a similar structure containing the Pro residue in their sequences [8,42,126]. Additionally, Cermeño et al. reported that compared between Ile (I) and Lue (L) residue, Ile containing peptides had higher bioactivities including DPP-IV inhibition [52]. In addition, the presence of hydrophobic residues at the C-terminal sequences have also been recognized in numerous DPP-IV inhibitory peptides reported previously. Therefore, the C-terminal position also enhances the DPP-IV inhibitory activity of peptide but to a lesser degree when compared with the N-terminal [2,127,128].

#### 3.6.2. Inhibition Modes of DPP-IV Inhibitory Peptides

**Figure 6:**
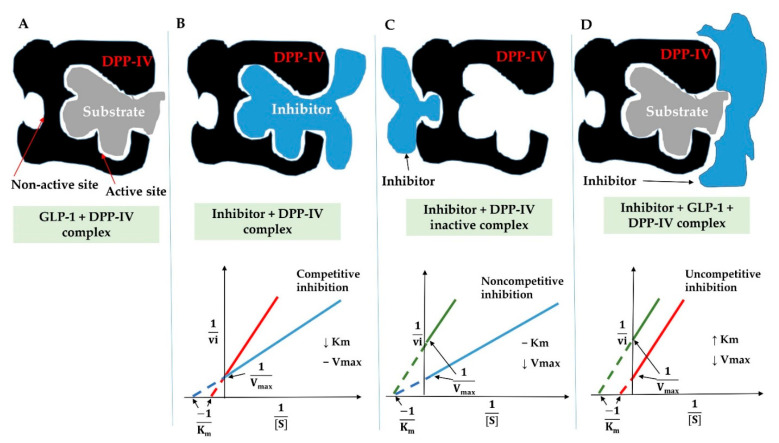
Inhibition modes of DPP-IV inhibitory peptides. (**A**) The complexion of the natural substrate (GLP-1) of DPP-IV and DPP-IV enzyme. DPP-IV inhibitors prevent DPP-IV enzymes from cleavage substrates through binding to active sites of DPP_IV as (**B**) competitive inhibition presenting via the Lineweaver-Burk plot results with a stable Vmax value and decreasing Km value based on the inhibitor concentrations; interaction at the secondary binding sites of DPP-IV as (**C**) non-competitive inhibition that performs a constant Km value and reducing Vmax value; and (**D**) uncompetitive inhibition that shows increasing Km value and decreasing Vmax value depending on the levels of inhibitors.

Based on the Lineweaver–Burk plot results, inhibition modes of DPP-IV peptide activity are classified into competitive, non-competitive, uncompetitive, and mixed-type inhibition [129]. Combining kinetic study and molecular docking of DPP-IV peptides can enhance the understanding of the site of interaction between DPP-IV enzymes and peptides, also revealing the DPP-IV active sites. First, competitive peptides can bind directly to the active sites of DPP-IV and can prevent substrate (GLP-1) binding with the enzyme (Figure 6A,B). The linear regression curves are established from the intersection of different concentrations of inhibitors (the horizontal axis) with the enzymatic reaction rate (the vertical axis). As indicated in the Lineweaver-Burk double reciprocal graph, these peptides are considered as the competitive activity type when the Km value ascends according to the inhibitor concentration, while the Vmax value is stably maintained (Figure 6B). All of the strongest DPP-IV inhibition peptides containing IPI (Diprotin A), VPV, and VPL (Diprotin B) are examples of competitive inhibitors [8,130]. Therefore, competitive peptides, the most popular inhibitor, play an outstanding role in investigating food-derived novel peptides possessing a great DPP-IV inhibition activity (Table 1). The high frequency of specific residues (P or A) at the second position of the N-terminal sequences was recognized in almost all the competitive DPP-IV peptides derived from various dietary proteins such as yolk egg turtle, milk, and salmon [2,3,8,48,80]. Unlike the competitive inhibitors, the remaining modes (non-competitive, uncompetitive, and mixed-type) can only interact outside of the active sites of DPP-IV (Figure 6C,D). Specifically, non-competitive inhibitors can interact with secondary binding sites and create the inactive complex, therefore reducing the catalytic activity of DPP-IV (Figure 6C). In the uncompetitive DPP-IV inhibition mode, inhibitors only bind to the substrate–enzyme complex, leading to decreased enzyme activity and ensuring the substrate is out of the enzyme active sites (Figure 6D). The presence of Trp residue at their N-terminal in peptide sequences significantly changes the modes of DPP-IV inhibitors. For example, most Trp containing dipeptides were determined as non-competitive inhibitors of DPP-IV; however, all 19 Trp-Arg-Xaa tripeptides displayed the uncompetitive type inhibition [7,42,131,132]. Generally, the mixed inhibition mechanism has been covered by and formed from competitive and uncompetitive inhibitions. To date, a significant number of peptides were determined as non-competitive, uncompetitive, or mixed-type modes of DPP-IV inhibitors [2,3,35,42,48,124].

#### 3.6.3. The Stability of the DPP-IV Inhibition Peptides

The DPP-IV inhibition peptides have also been classified as true, substrate, or prodrug inhibitors based on the peptide stability under pre-incubation with the DPP-IV enzyme [121,133]. The results of peptide hydrolysis were regularly identified using liquid chromatography–tandem mass spectrometry (LC-MS/MS) [2,42,48]. True inhibitors are not hydrolyzed by the DPP-IV activity and retain the effect of DPP-IV inhibition activity. Conversely, prodrug and real substrate types are cleaved after incubation time with DPP-IV enzyme and respectively releasing products with more and less potent inhibitors than the original molecule. The high frequency of the Pro/Ala amino acid in the second position at their N-terminal is the preferential characteristic feature of substrate inhibitors towards the DPP-IV enzyme. Diprotin A and B (IPI and VPL) are typical examples of substrate DPP-IV inhibitors [130]. However, peptide Leu-Pro-Leu-Pro-Leu (LPLPL) that possesses the preferred structure of the DPP-IV inhibitory substrate was reported as a prodrug DPP-IV inhibitor [133]. In addition, several tripeptides, in particular APA, APF, APR, IPA, KPA, FPI, and WPI, were not hydrolyzed by DPP-IV after 30 min of incubation [42]. Until now, almost all food-derived peptides are known to act as substrate DPP-IV inhibitors [2,42,48,121,134].

#### 3.6.4. Quantification of Peptides

Quantification measurement of target peptides is commonly determined using a method known as either the selected reaction monitoring (SRM) or multiple reaction monitoring (MRM) mode during LC-MS/MS analysis [135]. The quantity of selected peptide abundance in parent proteins is monitored accurately based on precursors (intact peptide ions) and product ions (corresponding specific fragment ions) of the peptide sequence, leading to high precision, sensitivity, and throughput identification. Moreover, MRM can provide an analysis of multiple peptides in a single LC-MS run. Several studies reported on the application of the MRM method to measure the peptide amount present in food proteins like egg and plants [98,136,137,138,139].

#### 3.6.5. Molecular Docking

The virtual investigation of the interactions between the DPP-IV receptors and the potential DPP-IV inhibitors is widely carried out using molecular docking. Additionally, molecular docking can reveal the binding modes and residues at the interacted positions, therefore allowing for the prediction of peptides’ DPP-IV inhibition activity [140,141,142]. In molecular analysis, the lower CDOCKER energy value (known as a molecular dynamic simulated-annealing-based algorithm) of interaction between a peptide and the target DPP-IV suggests a higher probability of a potential DPP-IV inhibitory peptide. Generally, peptide ligands corresponding with various action modes (as competitive, non-competitive, uncompetitive, and mixed-type modes) will give a different value for binding scores. DPP-IV has some interaction pockets in which peptide ligands can bind, such as a hydrophobic S1 pocket (Tyr631, Val656, Trp659, Tyr662, Tyr666, and Val711), a charged S2 pocket (Arg125, Glu205, Glu206, Phe357, Ser209, Val 207, and Arg358), and a catalytic triad (Ser630, Asp708 (Asn710), and His740) [122,141,143,144]. The relationship between ligands and receptors is commonly built through several interactions containing hydrogen-bonds, van der Waals forces, and charge or polar interactions. For docking simulation, various DPP_IV crystal conformations with different codes according to the compounds presented in the DPP_IV complex are selected from the protein data bank (PDB) (Table A3). Regularly, the provided DPP-IV complex depends on corresponding to peptides that require to evaluate via molecular docking [122]. For instance, Nongonierma et al. used porcine DPP-IV in complex with a peptidomimetic inhibitor (PDB code: 1ORW) for assessing 25 synthesized peptides by docking investigations, consistent with an in vitro experiment using the porcine DPP_IV enzyme [42]. Additionally, the DPP_IV structure (PDB code: 1WCY, the human DPP_IV complex with diprotin A) was chosen in docking studies for DPP-IV inhibitory peptides derived from milk protein [145], *Chlorella vulgaris* [146], and *Ruditapes philippinarum* [89], and matched through using diprotin A as a positive control in the DPP-IV assay. Moreover, the complexion of human DPP_IV with a cyclohexalamine inhibitor (PDB code: 2P8S), HL1 (PDB code: 5J3J), the long-acting inhibitor Omarigliptin (MK-3102) (PDB code: 4PNZ), and inhibitor 3 (PDB code: 5Y7H) was performed in studies of hen egg-white [41], hen egg [30], millet protein [38], and Atlantic salmon *(Salmo salar)* skin [3], respectively. Recently, Nongonierma et al. and Wang et al. reported that the binding sites of porcine DPP-IV and that of the human enzyme were highly conserved, specifically recognized at the S1 pocket, S2 pocket, and catalytic triad [41,42]. Therefore, the general mechanism for in vitro assay of DPP-IV inhibition activity of peptides derived from food hydrolysate could also be exposed using the DPP-IV enzyme of porcine. So far, numerous peptides derived from food hydrolysates were deeply investigated at a molecular level using various available docking software [4,30,38,80].

Interestingly, some peptides that showed a higher binding free energy score with high DPP-IV inhibitor activity were sourced from egg protein, milk, and salmon hydrolysates [3,41,127,145]. For instance, the lower interaction score of several peptides to DPP-IV, comparative to that of IPI (diprotin A), indicated that peptides could bind with the DPP-IV enzyme to form more stable complexes, promising better DPP-IV inhibitory activity [42,124,127,144,145]. However, according to in vitro results, they possessed lower DPP-IV inhibitory activity and some of them could not inhibit DPP-IV. Moreover, peptide YYGYTGAFR released from salmon skin collagen hydrolysate showed a lower binding free energy as compared with the remaining peptides LDKVFR and VLATSGPG, but LDKVFR achieved the highest DPP-IV inhibition activity with the IC_50_ value of 91.24 µM (Table 1) [3]. LDKVFR interacted with the DPP-IV enzyme through hydrogen bonds and hydrophobic interaction at pocket 2 (Glu205, Glu206, Ser209, Arg358, and Phe357), consistent with the kinetic study presented as a competitive peptide. All DPP-IV residues formed with VLATSGPG and YYGYTGAFR were not observed in the active binding sites, corresponding with the mode of action as non-competitive and mix-type modes, respectively. DPP-IV inhibitory peptides derived from *Ruditapes philippinarum* hydrolysate are another example that expresses the difference between docking prediction and in vitro results [89]. The docking results indicated that the DPP-IV inhibitory activity of peptides was reduced following AGDDAPR, LAPSTM, FAGDDAPRA, and FLMESH. However, the in vitro analysis of synthetic peptides showed that LAPSTM possessed the best DPP-IV inhibitory activity with IC_50_ values of 140.82 µM as compared to the remaining peptides FAGDDAPR, FAGDDAPRA, and FLMESH with IC_50_ indexes of 168.72 µM, 393.30 µM, and >500 µM, respectively. The Lineweaver–Burk double-reciprocal plot expressed that LAPSTM acts as a competitive-type inhibition mode for the DPP-IV enzyme, meaning this peptide can interact with active sites of DPP-IV. Correspondingly, LAPSTM was detected to bind with Try662 in the S1 pocket; with Arg125, and Ser209 in the S2 pocket; and with Ser630 in the catalytic triad. Docking analysis and kinetic results indicated that FAGDDAPR, FAGDDAPRA, and FLMESH displayed as mix-type inhibition modes, meaning they can bind to active sites and outside of the catalytic center of DPP-IV. Recently, Wang et al. reported that hen egg white-derived peptides possessed the potential DPP-IV inhibitory activity using in vitro assay and docking simulation [41]. X-ray crystal structures of human DPP-IV (PDB codes 3Q8W, 3F8S, and 5T4B) were docked to peptides IRDLLER, YAEERYP, and IRNVLQPS. Three peptides can interact with DPP-IV not only by hydrogen bonds but also by charge interactions and π-π interactions. Peptide IRDLLER (IC_50_ value of 186.23 µM) showed higher DPP-IV inhibitory activity than YAEERYP (IC_50_ value of 340.62 µM) and IRNVLQPS (IC_50_ value of 598.28 µM). According to the docking analysis, IRDLLER was predicted to form more hydrogen bonds and active sites in the S2 pockets than the remaining peptides, therefore hydrogen bonds and the S2 active sites may significantly contribute to the DPP-IV inhibitory activity of these peptides. The combination of molecular docking and in vitro experiments is necessary to have a better understanding of the bioactive peptides.

### 3.7. The Specific In Vitro Models of GLP-1 Secretion Studies

Nowadays, concerns about the safety and efficiency of drug diabetic treatment from food-derived bioactive peptides in humans have obviously grown. Therefore, leading in vitro and in vivo models are carefully selected to carry out examinations with an abundance of evidence and verification before such peptides are used in patients. In most of the current investigations, the DPP-IV inhibitory abilities of peptides are widely assessed in vitro using enteroendocrine cells located in the gut epithelium. The enteroendocrine cell lines commonly used to study mechanisms of glucagon-like peptide (GLP-1) secretion are GLUTag, NCI-H716, and STC-1 cells [147,148,149,150]. Such cell lines are widely used because they are easy to maintain in culture and useful for cell biological studies. All three cell lines are sourced from carcinomas with a slight difference. The cells of NCI-H716 were originally derived from the ascite fluid of a human colorectal adenocarcinoma, while GLUTag and STC-1 cells were developed from colonic tumors of the large bowel and small intestine of mice, respectively [151,152]. Cholecystokinin is contained and highly expressed in GLUTag cells, evident in low amounts in STC-1 cells but not available in NCI-H716 cells. No insulin or pancreatic polypeptide is secreted in GLUTag and STC-1 cells [148]. The NCI-H716 cell line releases the largest amounts of the expressed peptides as compared with GLUTag and STC-1 cells. However, GLUTag and STC-1 cells release more varying peptides [148]. The STC-1 cells have a noble potential to produce higher volumes of GLP-1 and DPP-IV than the GLUTag cells [149]. Until now, the NCI-H716 cell line had characteristics of endocrine cells and was currently the only human model available for studying the regulation of GLP-1 [151]. These cell lines have limitations that make them far from optimal models. For example, their preformation has a slight modification from native L-cell characteristics such as the lack of polarization (apical vs. basolateral surfaces), releasing some different peptides with standard L-cell products (GIP, somatostatin, and glucagon) [148]. Furthermore, the secretion of GLP-1 in these cells has affected several conditions such as derived hydrolysate on enzyme digestion or free amino of peptides (see more in Table A4).

Several research studies have conflicting data regarding the comparison of GLP-1 secretion results of intact proteins (non-hydrolysate proteins) and hydrolysate ones (enzyme digested proteins). In the sodium caseinate case, Y. Komatsu et al. identified a digested protein that has a positive effect on the GLP-1 production using the GLUTag cell line [153]. However, the results in Geraedts et al. indicated an opposite trend whereby intact sodium caseinate significantly improved GLP-1 secretion from STC-1 cells [154]. Furthermore, the effect of casein using the STC-1 cell line also showed a different tendency in the release of GLP-1 in other research [155]. Results of O’ Halloran et al. are in agreement with the increased GLP-1 secretion when compared to digested casein [156]. In contrast, Gillespie et al. reported that β-casein hydrolysate digested with chymotrypsin or trypsin (exception of pepsin) did not stimulate GLP-1 release [155]. Another previous study showed that whey protein hydrolysate and peptides have been found to inhibit DPP-IV and α-glucosidase [157,158], but only non-digested whey effectively induced GLP-1 release [159,160]. Contrarily, the study results of T. S. Moya et al. described that hydrolysate of digested whey produced higher GLP-1 secretion than intact protein [161]. Collectively, it can be speculated that the release of GLP-1 activity may be directly related to the peptide profile of hydrolysate that is influenced by the difference in the digestion conditions.

To date, only a few peptide sequences derived from bovine hemoglobin, casein, egg white, frog skin, and bacteria have been demonstrated to exhibit GLP-1 releasing effects in NCI-H716 [162,163], STC-1 [33,164,165], and GLUTag cell lines [153,166,167,168], and in murine primary cultures [169]. Recently, M. Santos-Hernández published a paper regarding the fact that egg white proteins only had a modest effect in inducing GLP-1 production, while peptides (except free amino acids) released a significant amount [33]. The molecular weight fractions derived from enzymatically digested proteins are related to this activity, showing that small fragments (smaller than 3 kDa in size) of casein hydrolysate increased GLP-1 secretion in STC-1 cells [156]. Small peptides may be responsible for GLP-1 secretion bioactivity but the lengths of peptides for the stimulation of maximal GLP-1 production are continuously being discovered [153,156,169]. M. Santos-Hernández also indicated that only two egg white peptides (PEL (3 amino acids) and RVASMASEKM (10 amino acids)) significantly released GLP-1 [33]. In addition, amino acids such as glutamine, alanine, serine, phenylalanine, tryptophan, lysine, and valine also remarkably enhance GLP-1 secretion [156,170,171,172]. Generally, the length, characteristic, and sequence of peptides play notable roles in improving this secretagogue effect.

### 3.8. In Vivo Effect of Food-Derived DPP-IV Inhibitory Peptides

#### 3.8.1. In Vivo Animal Studies

In most research studies, animal models mimicking the pathology of human T2D always play an important role in providing more evidence to validate the effects of food-derived DPP-IV inhibitors. The high-fat diet/streptozotocin-treated (HFD/STZ) rat model is one of the popularly typical examples of an experimentally-induced animal model of diabetes. This model is induced by a combination of a high-fat diet and STZ treatment that cause glucose resistance. Pathological changes and clinical features recognized in animals after STZ treatment resemble those of human diabetes; therefore, STZ is the most preferable chemical used to induce diabetes in animals [173]. Recently, the STZ-induced diabetic models have been successfully performed in animals such as mice, rats, monkeys, guinea pigs, hamsters, dogs, and cats. However, the different species, strain, gender, and age of the animal also significantly affects the diabetogenic activity of STZ. Mice models have been commonly used to study the effect of DPP-IV inhibitors on diabetes disease due to several of their benefits, for instance, concerning the ease of handling, small size, and cost-effectiveness; however, it has a trend-forward to human type 1 more than T2D [173]. Moreover, the CD-1 and C57BL/6 murine models have also been reported as more sensitive to STZ toxicity. Therefore, rat models have been widely and preferably used because their large features permit more and easier observation of the display of these kinds of invasive procedures as compared to the mice model. The source of the animal is an important factor that determines the success of animal diabetes models produced by STZ treatment. While Wistar–Kyoto rats revealed their resistance to the action of STZ, both Wistar and Sprague Dawley rats exposed their sensitivity to STZ. The resource of a high lipoprotein lipase pool in the Wistar–Kyoto strain may be the main reason for the above difference. In addition, the resistance to the diabetogenic action of STZ is also recognized in rabbits. In addition, the gender or age of animals likewise plays a notable role in making the successful diabetes model. Interestingly, the difference in hormones between the genders may be the reason why male rodents are more responsive according to the display of diabetes symptoms released from STZ activity as compared to females [174]. It is also intriguing that mice or rats of eight weeks of age have increased mortality risks after STZ administration than older animals [175].

The number of publications about the food-derived components that inhibit DPP-IV action is still modest. Nonetheless, findings from the few animal studies regarding the effects of the intact proteins, enzyme digestion proteins (hydrolysate), and peptides with diabetic models are promising. Most of the extract of proteins (non-hydrolysate proteins) with proven anti-diabetes effects in vivo are derived from medicinal plants such as *H. rosa Sinensis* leaves [50], *Rhazya stricta* root [176], *Terminalia arjuna* [51,177], *Pueraria tuberosa* [178], Amaranth grain [179], and Grapeseed [180], with one animal source exception of sea urchin gonads [56]. All of these demonstrated inhibitory activity against DPP-IV enzymes, corresponding to an increase in the secretion of GLP-1. In addition, the reduction of serum glucose levels has also been recognized in both urchin gonad tissue extracts [56] and the extract of *H. rosa-Sinensis* leaves [50] (Table A5).

To date, only several animal studies have evaluated the efficacy of DPP-IV inhibitory hydrolysates such as porcine skin [181], fish skin [55,182], rice bran [183], zein corn [184], and sodium caseinate [185] hydrolysate with significant positive results. In these research studies, the commercial proteases commonly used to release hydrolysate are flavourzyme [181,182,186], papain [183,184], and pepsin [183] due to their potent GLP-1 secretory activity in vitro and in vivo (flavourzyme and papain), and their partial simulation of gastric digestion (pepsin). Additionally, oral administration is preferred over subcutaneous administration in almost all animal models due to advantages including easier management, less complex handling, painless, and safety features. Administration time with hydrolysates ranges from four weeks to around eight weeks according to the administered concentration under daily control. Moreover, the treatment with express hydrolysate reduces in-plasma DPP-IV activity, which has been associated with an improvement in both active and total GLP-1 concentration. Increasing GLP-1 levels correlate with changes in enhanced insulin and lower glycemia.

In addition to hydrolysate, dietary peptides with in vitro DPP-IV inhibitory activity have been reported to induce GLP-1 secretion in vivo. The treatment of ß-casein-derived peptide LPQNIPPL identified in gouda-type cheese was discovered to potentiate the DPP-IV inhibitory activity in animal models [187]. Rats that were orally administered with octapeptides showed a notable reduction in the post-prandial plasma glucose area under the curve during an oral glucose tolerance test compared with that of the control group (without peptide). However, the plasma insulin levels were not significantly different between the presence and absence of peptide groups. In that study, the plasma DPP-IV inhibitory activity was not determined. Similarly, the anti-diabetic effects of peptides produced from Chinese black tea in vivo were also recently reported [188]. A peptide (AGFAGDDAPR) with a positive DPP-IV inhibitory effect (in vitro) administered in diabetic mice significantly enhanced the concentration of GLP-1 in the blood as well as dramatically the increased insulin level compared to that in the control diabetic animals after 57 days of peptide treatment. Moreover, immunohistochemistry also revealed decreasing pancreatic alpha-cell proliferation and improving pancreatic beta-cell function.

The data discussed highlighted food-derived DPP-IV inhibitory components that have the potential to be a functional food for anti-hyperglycemia. Therefore, in bioactive peptides studies, the evaluation of the peptide characteristics to inhibit DPP-IV activity in vivo is necessary after screening for their DPP-IV inhibition ability in vitro. However, adequate analysis of peptides based on their safety, efficacy, and potency should also be carried out in more detailed clinical studies before market introduction.

#### 3.8.2. Clinical Studies

So far, several human studies have evaluated the anti-diabetes efficiency of food-protein hydrolysates or peptides. Milk and fish protein hydrolysates containing bioactive peptides are commonly used and the most applied [32,189,190,191,192,193,194]. In sixty male and female diabetes patients treated orally and daily with 10 mg of fish collagen peptides (properties DPP-IV inhibitors), a significant reduction in fasting blood glucose and transition of glycosylated hemoglobin to normal levels was observed after three months of management [193]. Recently, the administration of a twice-daily dose of 8.5 g of a casein protein hydrolysate to patients with gestational diabetes moderately decreased plasma glucose levels, indicating an improved diabetes status [194]. Moreover, the control of postprandial glucose levels was extremely improved by supplementing 1.4 g per day of whey hydrolysates [189]. In conclusion, the dietary protein hydrolysate-derived peptides are recognized as promising and are well-tolerated for prevention and cure in diabetic patients; however, further clinical studies should be carried out with longer intervention periods to confirm their efficiency.

## 4. Future Perspectives

Diverse active peptides can be uncovered from food-protein hydrolysates. In addition to DPP-IV inhibitory activities, some peptides also showed multiple health benefits. The direction of DPP-IV inhibitory peptide studies is concentrating on the increase of more effective hydrolysate proteins and peptides. However, there are several challenges to face in the discovery of novel peptides from food proteins, including optimal methods for the separation and purification of individual peptides from complex peptides; the low bioactivity efficiency of peptides; toxicity of peptides in human health; and determining the perfect cell and animal model for anti-diabetes activity studies. The cell cultures and small animal studies are essential to elucidate the efficiency of the current research to the human body and to discover the suitable dose of hydrolysate, peptides for maintaining control over diabetic characteristics in vivo. Furthermore, the quantification of the amount of hydrolysate from protein samples after enzymatic hydrolysis and the yield of potent peptides derived from their hydrolysate proteins are necessarily calculated to evaluate the capability of samples for application in industry.

The use of multiple functional bioactive peptides is the rapid tendency development in the study of food-protein hydrolysates/peptides based on the association of T2D with metabolic syndromes. The peptides released from whey protein hydrolysates, hen egg proteins, and ham byproducts are several examples of the dual effects of anti-diabetes (DPP-IV inhibition peptides) and antihypertension (ACE inhibition peptides) properties [1,30,83,195]. Moreover, peptides possessing three bioactivities such as ACE, α-glucosidase, and lipase inhibitory activities have been recognized in species of edible insects [13]. Interestingly, Valencia et al. reported that gliptin, a DPP-IV inhibitor, has potential to hinder SARSCoV-2 infection in macrophages, which seems useful for constraining the development of the virus [196].

According to the available literature, food-protein and food-derived peptides have proven their potential antidiabetic activity efficiency by their inhibition effect of the DPP-IV, α-glucosidase, and PTP-1B enzyme expression, leading to reduced hyperglycemia. Additionally, the strategies for discovering novel peptides are also of notable concern. Generally, promising peptides are mainly considered for their high biological effects, safety, and low cost-efficiency research. Although food-derived peptides contain numerous advantages that may advance their use as functional foods or pharmaceutical drugs, the examinations should be carried out carefully and sufficiently, and be combined with clinical trials. These studies are necessary to validate the efficacy and bioavailability of peptides.

## Figures and Tables

**Table 1:** Summary of recent publications concerning DPP-IV inhibitory peptides (2018–2020).

Precursor Protein	Sequence	IC_50_	Mode of Inhibition	Peptide Hydrolyzed by DPP-IV	Docking	Cell Model/In Vivo	Reference
Boarfish protein hydrolysate	IPVDM	21.72 µM	nd	nd	nd	BRIN-BD11 cells Caco-2 cell	[36]
	APIT	34.73 µM	nd	nd	nd		
	VPTP	38.93 µM	nd	nd	nd		
	GPIN	48.96 µM	nd	nd	nd		
	LPVYD	51.36 µM	nd	nd	nd		
	LPVDM	53.50 µM	nd	nd	nd		
	APLER	63.67 µM	nd	nd	nd		
	IPGA	66.37 µM	nd	nd	nd		
	GPSL	68.13 µM	nd	nd	nd		
	GPSI	73.15 µM	nd	nd	nd		
	APVP	79.10 µM	nd	nd	nd		
	VPDPR	90.37 µM	nd	nd	nd		
	APLT	91.10 µM	nd	nd	nd		
	APLT	115.27 µM	nd	nd	nd		
	MPAVP	116.27 µM	nd	nd	nd		
	GPGI	116.37 µM	nd	nd	nd		
	GPLN	126.51 µM	nd	nd	nd		
	PAVP	131.90 µM	nd	nd	nd		
	GPGL	154.12 µM	nd	nd	nd		
	LPGA	164.37 µM	nd	nd	nd		
	AALP	164.52 µM	nd	nd	nd		
	TPTV	251.58 µM	nd	nd	nd		
	AAIP	261.58 µM	nd	nd	nd		
	YPL(pS)L	261.46 µM	nd	nd	nd		
	TPGI	282.16 µM	nd	nd	nd		
	TPGL	297.41 µM	nd	nd	nd		
	YPII(pS)	300.67 µM	nd	nd	nd		
	YPIL(pS)	302.47 µM	nd	nd	nd		
	ISAP	393.88 µM	nd	nd	nd		
	YPL(pT)V	511.47 µM	nd	nd	nd		
	YPLV(pT)	508.37 µM	nd	nd	nd		
α-lactalbumin-rich whey protein	LDQWLCEKL	131 μM	Non-competitive	nd	nd	nd	[35]
	EQLTKCEVFR	883 μM	nd	nd	nd	nd	
	KILDKVGINYWLAHK	930 μM	nd	nd	nd	nd	
	ILDKVGINYWLAHK	456 μM	nd	nd	nd	nd	
	VGINYWLAHK	765 μM	Mixed-type	nd	nd	nd	
Atlantic salmon (Salmo salar) skin	YYGYTGAFR	91.24 µM (0.1 mg/mL)	Mixed-type	nd	Yes	nd	[3]
	LDKVFR	231.95 µM (0.18 mg/mL)	Competitive	nd	Yes	nd	
	VLATSGPG	1.73 mM (1.21 mg/mL)	Non-competitive	nd	Yes	nd	
Porphyra dioica protein	YLVA	439.5 μM	nd	nd	nd	nd	[97]
Soft-shelled turtle yolk hydrolysate	VPGLAL	289.2 μM	Competitive	Yes	nd	nd	[2]
	LPSW	269.7 μM	Competitive	Yes	nd	nd	
	LPLF	463.6 μM	Competitive	Yes	nd	nd	
	WLQL	432.5 μM	Uncompetitive	No	nd	nd	
Egg white ovalbumin	CF	2.99 mM	nd	nd	nd	nd	[1]
	KM	2.22 mM	nd	nd	nd	nd	
	ELPF	9.92 mM	nd	nd	nd	nd	
	AM	2.79 mM	nd	nd	nd	nd	
	ADHPF	1.66 mM	nd	nd	nd	nd	
	LPR	1.43 mM	nd	nd	nd	nd	
	PR	4.11 mM	nd	nd	nd	nd	
	FR	2.47 mM	nd	nd	nd	nd	
	PRM	2.50 mM	nd	nd	nd	nd	
	GR	2.83 mM	nd	nd	nd	nd	
Hen egg proteins	ADF	16.83 mM	nd	nd	Yes	nd	[30]
	MIR	4.86 mM	nd	nd	Yes	nd	
	FGR	46.22 mM	nd	nd	Yes	nd	
	CDR	24.49 mM	nd	nd	nd	nd	
Dark tea protein	MSLYPR	1.76 mM (1.35 mg/mL)	nd	nd	Yes	nd	[31]
	QGQELLPSDFK	3.08 mM (3.89 mg/mL)	nd	nd	Yes	nd	
Brewers’ spent grain	APLP	122.45 µM	nd	nd	nd	nd	[52]
	IPIPQ	75.70 µM	nd	nd	nd	nd	
	IPLQP	105.45 µM	nd	nd	nd	nd	
	IPVP	38.67 µM	nd	nd	nd	nd	
	IPY	52.15 µM	nd	nd	nd	nd	
	LAVP	98.76 µM	nd	nd	nd	nd	
	LPIA	45.07 µM	nd	nd	nd	nd	
	LPVP	105.25 µM	nd	nd	nd	nd	
	LPY	87.15 µM	nd	nd	nd	nd	
	PAIP	111.18 µM	nd	nd	nd	nd	
S. platensis protein	LRSELAAWSR	140.94 µM (167.3 µg/mL)	nd	nd	Yes	HepG2 cell	[123]
Camel whey protein	VPV	6.6 µM	Competitive	nd	nd	nd	[8]
	YPI	35.0 µM	Competitive	nd	nd	nd	
	VPF	55.1 µM	Competitive	nd	nd	nd	
	EPVK	330.1 µM	Competitive	nd	nd	nd	
	LAHKPL	239.7 µM	Competitive	nd	nd	nd	
	YPLR	360.1 µM	Competitive	nd	nd	nd	
Camel milk protein	ILDKEGIDY	347.8 µM	Mixed-type	No	nd	nd	[48]
	ILDKVGINY	321.5 µM	Mixed-type	No	nd	nd	
	ILELA	721.1 µM	Competitive	No	nd	nd	
	LLQLEAIR	177.8 µM	Competitive	No	nd	nd	
	LPVP	87.0 µM	Competitive	No	nd	nd	
	MPVQA	93.3 µM	Competitive	Yes	nd	nd	
	SPVVPF	214.1 µM	Mixed-type	Yes	nd	nd	
	YPVEPF	138.0 µM	Mixed-type	Yes	nd	nd	
Silver carp (*Hypophthalmichthys molitrix*) muscle hydrolysate	LLDLGVP	2665.46 µM	nd	nd	nd	nd	[96]
	AALEQTER	647.02 µM	nd	nd	nd	nd	
	ILYGDFK	2614.14 µM	nd	nd	nd	nd	
	KAVGEPPLF	1317.39 µM	nd	nd	nd	nd	
	GPAGPQGPR	842.10 µM	nd	nd	nd	nd	
Synthetic	PFP	1389.14 µM	Uncompetitive	nd	Yes	3T3-L1	[124]
	YPL	364.62 µM	Uncompetitive	nd	Yes		
	YPG	173.96 µM	Uncompetitive	nd	Yes		
Egg white	IRDLLER	186.23 μM	nd	nd	Yes	Caco-2 cell/Wistar male rats	[41]
	YAEERYP	340.62 μM	nd	nd	Yes		
	IRNVLQPS	598.28 μM	nd	nd	Yes		
Napin of rapeseed (Brassica napus)	PAGPF	135.70 μM	Mixed-type	nd	Yes	nd	[80]
	IPQVS	52.16 μM	Competitive	nd	Yes	nd	
	ELHQEEPL	78.46 μM	Mixed-type	nd	Yes	nd	
	KTMPGP	162.73 μM	Uncompetitive	nd	Yes	nd	
Analogs of ile-Pro-ile	APA	43.3 μM	Competitive	No	Yes	nd	[42]
	APF	65.8 μM	Competitive	No	Yes	nd	
	APR	119.7 μM	Competitive	No	Yes	nd	
	IPA	28.3 μM	Competitive	No	Yes	nd	
	KPA	74.5 μM	Competitive	No	Yes	nd	
	FPF	247 μM	Competitive	Yes	Yes	nd	
	FPI	45.2 μM	Competitive	No	Yes	nd	
	FPW	54.9 μM	Competitive	Yes	Yes	nd	
	IPF	47.3 μM	Competitive	Yes	Yes	nd	
	IPW	175.3 μM	Competitive	Yes	Yes	nd	
	WPF	159.8 μM	Mixed-type	Yes	Yes	nd	
	WPT	133 μM	Mixed-type	No	Yes	nd	
	WPW	120.1 μM	Mixed-type	Yes	Yes	nd	
Water-soluble extracts from natto	KL	159.84 μM (41.40 µg/mL)	nd	nd	nd	nd	[94]
	LR	2.08 mM (598.02 µg/mL)	nd	nd	nd	nd	

The pS and pT represent phosphorylation at Ser and Thr, respectively. Note: IC_50_, the DPP-IV half-maximal inhibitory concentration; nd, not determined; and Yes/No, with or without analyses experiments.

## Appendix A

**Table A1:** Food protein-derived with α-glucosidase inhibition peptides (2016–2020).

Source	Enzyme	Sequence	IC_50_	Reference
Quinoa hydrolysate	Gastrointestinal enzyme	IQAEGGLT	109.48 µM	[53]
Almond oil manufacture residue	Prote Ax and protease M	WH	17.03 µM (17.03 μmol/L)	[37]
		WS	2.47 µM (24.71 μmol/L)	
Rational in silico design		SVPA	4.56 mM	[76]
		SEPA	0.79 mM	
Sprouted quinoa yoghurt	*Lactobacillus casei*	VAHPVF	13.47 µM (9 ppm)	[77]
		LAHMIVAGA	12.37 µM (10.9 ppm)	
		KSFGSSNI	22.07 µM (18.5 ppm)	
		KDLQL	26.01 µM (16 ppm)	
		MIKLRSTAKN	12.15 µM (14.1 ppm)	
Soy protein	Alkaline proteinase	LLPLPVLK	237.43 µM (237.43 μmol/L)	[75]
		SWLRL	182.05 µM (182.05 μmol/L)	
		WLRL	162.29 µM (162.29 μmol/L)	
Silkworm pupae	In silico digestion	QPGR	65.6 µM (65.8 µmol/L)	[28]
		SQSPA	20 µM (20 µmol/L)	
		QPPT	560 µM (560 µmol/L)	
		NSPR	205 µM (205 µmol/L)	
Dark tea protein	Trypsin	TAELLPR	0.53 mM (0.43 mg/mL)	[31]
		CGKKFVR	0.62 mM (0.52 mg/mL)	
		AVPANLVDLNVPALLK	0.62 mM (1.03 mg/mL)	
		VVDLVFFAAAK	33.95 µM (0.04 mg/mL)	
Dibble insects	Simulated intestinal digestion (pancreatin and bile extract)	IIAPPER	28.79 µM (22.86 µg/mL)	[13]
		LAPSTIK	62.63 µM (45.60 µg/mL)	
		KVEGDLK	23.34 µM (18.37 µg/mL)	
		NYVADGLG	25.24 µM (20.37 µg/mL)	
		AAAPVAVAK	13.71 µM (10.92 µg/mL)	
		AGDDAPR	27.81 µM (19.47 µg/mL)	
		GKDAVIV	22.77 µM (15.94 µg/mL)	
		AIGVGAIER	14.75 µM (13.04 µg/mL)	
		FDPFPK	7.94 µM (5.95 µg/mL)	
Spanish dry-cured ham		AEEEYPDL	5.58 mM	[34]
		LGVGG	6.36 mM	
		GGLGP	8.71 mM	
		EA	17 mM	
		PP	18.3 mM	
		VE	22.17 mM	
		PE	25.05 mM	
		AD	25.66 mM	

**Table A2:** Recently reported examples of methods on DPP-IV inhibitory peptides fractionation and purification (2016–2020).

Protein Source	Enzymatic Treatment	IC_50_ DPP-IV of Hydrolysate	Fraction Method	Materials	Reference
Bovine α-Lactalbumin hydrolysates	Alcalase		1. Gel filtration chromatography 2. Reversed-phase high-performance liquid chromatography (RP-HPLC)	1. Sephadex G-25 column (2.5 cm × 70 cm) 2. C18 RP-HPLC (4.6 mm × 250 mm, 5 µm)	[4]
Millet protein hydrolysates	α-amylase and cellulase		1. Gel filtration chromatography 2. RP-HPLC	1. Sephadex G-25 column (2.5 cm × 70 cm) 2. C18 RP-HPLC (250 × 4.6 mm, 5 µm)	[38]
Boarfish protein hydrolysate	Alcalase 2.4L and flavourzyme 500L	1.46 ± 0.06 mg/mL	Semi-preparative reverse phase-high performance liquid chromatography (SP-RP-HPLC)	C18 semi-preparative column (250 × 15 mm I.D., 10 µM)	[36]
α-lactalbumin-rich whey protein	Trypsin	2.88 ± 0.150 mg/mL	Gel filtration chromatography	Superdex peptide 10/300 GL	[35]
Atlantic salmon (Salmo salar) skin	Trypsin	1.54 ± 0.06 mg/mL	1. Gel filtration chromatography 2. SP-RP-HPLC	1. Sephadex G–25 gel column (1.6 cm × 60 cm) 2. C18 column (250 × 4.6 mm, 5 µm)	[3]
A Porphyra dioica protein	Alcalase and flavourzyme		SP-RP-HPLC	C18 semi-preparative column (250 × 15 mm I.D., 10 mm)	[97]
Soft-shelled turtle yolk hydrolysate	Pepsin, trypsin, and α-chymotrypsin	1.017 ± 0.0356 mg/mL	1. Ultrafiltration 2. RP-HPLC 3. Strong cation exchange (SCX) chromatography	1. <3 kDa MWCO 2. C18 column (10 mm × 250 mm; 5 µm)	[2]
Dark tea protein	Trypsin		Ultrafiltration		[31]
Brewers’ spent grain	Pepsin		1. Gel permeation-high performance liquid chromatography (GP-HPLC) 2. Reversed phase (RP-)-ultra-high performance liquid chromatography (UPLC)	1. TSK G2000 SW separating column (600 × 7.5 mm ID) connected to a TSKGEL SW guard column (75 × 7.5 mm) 2. C18 column (2.1 mm × 50 mm × 1.7 µm)	[52]
Camel whey protein	Trypsin	Between 0.55 ± 0.05 and 1.52 ± 0.16mg/L	1. UPLC 2. GP-HPLC	1. C18 column (2.1 mm × 50 mm × 1.7 µm) 2. TSK G2000 SW separating column (600 × 7.5 mm) connected to a TSKGEL SW guard column (75 × 7.5 mm)	[8]
Bambara bean protein hydrolysates	Alcalase and thermolysin	1.73 mg/mL	1. RP-HPLC 2. Gel permeation chromatography with UV/fluorescence detection		[91]
Camel milk protein hydrolysate	Alcalase, bromelain, and papain		RP-HPLC	C4 column (3.4 μm HPLC Column)	[49]
Silver carp (*Hypophthalmichthys molitrix*) muscle hydrolysate	Neutrase		1. Ultrafiltration 2. Gel filtration chromatography 3. SP-RP-HPLC	1. <3 kDa MWCO 2. Sephadex column (0.6 × 100 cm) 3. C18 column (4.6 × 250 mm)	[96]
Egg white	Simulated digestion (pepsin and trypsin)		RP-HPLC	C18 column (250 × 4.6 mm, 5 µm)	[41]
Napin of rapeseed (Brassica napus)	Alcalase and trypsin	0.68 mg/mL	1. Ultrafiltration 2. High-performance size-exclusion chromatography (SEC-HPLC)	1. <1 kDa MWCO 2. TSK gel 2000 SWXL column (300 × 7.8 mm)	[80]
Tropical banded cricket (*Gryllodes sigillatus*) proteins	Protamex^TM^	Between 0.40 ± 0.03 and 1.01 ± 0.07 mg/mL	1. Ultrafiltration 2. Gel permeation high performance liquid chromatography (GP-HPLC)	1. <1 kDa MWCO 2. TSK G2000 SW separating column (600 × 7.5 mm) connected to a TSKGEL SW guard column (75 × 7.5 mm)	[9]
Water-soluble extracts from natto		5.35 ± 0.27 mg/mL	1. Ultrafiltration 2. Gel filtration high-performance liquid chromatography (HPLC)	1. <3 kDa MWCO 2. Superdex peptide 10/300 GL	[94]
Wheat gluten hydrolysate	Pepsin and corolase PP	Between 0.24 ± 0.02 and 0.66 ± 0.06 mg/mL	1. Ultrafiltration 2. GP-HPLC 3. RP_UHPLC	1. <1 kDa MWCO 2. TSK G2000 SW separating column (600 × 7.5 mm) connected to a TSKGEL SW guard column (75 × 7.5 mm) 3. C18 column (2.1 mm × 50 mm × 1.7 µm)	[81]
Bighead carp (hypophthalmichthys nobilis) muscle hydrolysate	Pepsin		1. Ultrafiltration 2. Gel filtration chromatography 3. SP-RP-HPLC	1. <3 kDa MWCO 2. Sephadex G-15 column (0.6 × 100 cm) 3. C18 column (4.6 × 250 mm)	[90]
Ruditapes philippinarum hydrolysate	Papain	672.12 µg/mL	1. Ethanol precipitation 2. RP-HPLC	1. 2. C18 column (5 µm, 19 × 150 mm)	[89]
Barbel protein hydrolysate	Alcalase^®^	1.94 mg/mL	1. Size exclusion chromatography (SEC) 2. RP-HPLC 3. RP-HPLC	1. Superdex peptide HR 10/30 2. TRACER-Excel 1200 3. C18 column (2.1 × 150 mm, 2.7 m)	[92]
Silver carp protein (SCP) hydrolysates	Neutrase	1.12 mg/mL	1. Ultrafiltration 2. Thin-layer chromatography (TLC) fractionation 3. RP-HPLC	1. <3 kDa MWCO 2. Silica gel 60 TLC plate 3. C18 column (250 × 4.6 mm, 5 µm)	[54]
Sardine pilchardus protein	Flavourzyme	1.83 ± 0.05 mg/mL	Size exclusion chromatography (SEC)	Superdex peptide 10/300GL column	[93]
Mare Whey Proteins	Papain	0.18 mg/mL	1. Gel filtration chromatography 2. RP-HPLC	1. Sephadex G-25 column (2.5 cm × 70 cm) 2. C18 column (250 × 4.6 mm, 5 µm)	[79]
Quinoa (Chenopodium quinoa Willd.)	Pepsin and pancreatin	0.31 ± 0.01 mg/mL	1. Ultrafiltration 2. RP-HPLC	1. <5 kDa MWCO 2. Hi-pore reversed phase RP-318 (250 21.5 mm) column	[53]

**Table A3:** Summary of the interaction of peptide—DPP_IV proteins using the molecular docking analysis.

Protein Data Bank (PDB) ID	Software	Peptide	Binding Sites	Reference
1WCY	Discovery studio 4.0	FAGDDAPR,	Arg125, Arg358, Lys554, Phe357	[89]
		LAPSTM	Ser630, Arg125, Ser209, Try662, Tyr666, Arg125	
		FAGDDAPRA	Ser209, Tyr547, Tyr585	
		FLMESH	Arg125, Arg358	
1WCY	Discovery studio 4.0 software	VPW	Arg358, Tyr662, Ser630	[146]
		IPR	Glu205, Glu206, Tyr662	
3Q8W, 3F8S, and 5T4B	Discovery studio (DS) 2017 client software	IRDLLER	Glu205, Val207, Arg125, Arg429, Val546, Tyr585, Trp627, and Tyr752, Glu206, Arg125, Arg356 Arg429, and Asp545, Trp629, Phe357	[41]
		YAEERYP	Glu206, Arg356, Arg358, Glu361, Tyr456, Gln553, Asp556, Arg358, Arg429, Asp556, Arg356, Lys554 and Arg560	
		IRNVLQPS	Arg125, Phe357, Arg560, Ser630, and Tyr666	
5J3J	Discovery studio (DS) 2017 client software	ADF	Glu205, Glu206, Arg125, Arg358, Phe357	[30]
		MIR	Phe357, Arg358, Glu205, Glu206, Arg125	
		FGR	Tyr547, Tyr631, Val656, Phe357, Trp659, Arg669, Tyr666, Tyr662, Glu205, Glu206, Asp663, Arg125, His126	
5Y7H	Auto dock tools	YYGYTGAFR	Phe461, Ile407, Glu361, Gln469, Leu60, Ser59, Arg61, Asp104	[3]
		LDKVFR	Glu408, Arg356, Tyr585, Glu205, Glu206, Ser209, Ile470, Gly406, Arg358, Glu361, Ile405, Cys551, Phe357, and Arg669	
		VLATSGPG	Val459, Ser59, Ser460, Arg61	
4PNZ	Maestrov software	ELKDLKGY	Arg125, Ser630, Asn710, Tyr547, Ser209, Val207, Glu205, Glu361, Glu408, Arg429, Tyr456, and Asp556, Arg125, Glu361, Glu408, and Arg429, Tyr666	[4]
		ILDKVGINY	Arg356, Arg357, Arg358, Glu408, Arg429, Arg560, Arg358, Arg125	
4PNZ	Maestrov software	NDWHTGPLS	Tyr666, Ser630, Ser209, Arg125, Glu205, Glu206, Tyr662, Tyr456, Arg560, Arg429, Tyr547	[38]
		TYPHQQPPILT	Glu206, Ser630, Arg125, Arg358, Lys554, Tyr547, Ser662, His126, Ser209, Glu408	
4PNZ	Discovery studio 4.0	PAGPF	Asn740, Ser630	[80]
		IPQVS	Asn740, Arg125, Glu206	
		ELHQEEPL	Arg125, Glu206, Arg358	
		KTMPGP	Tyr547, Arg358	

**Table A4:** In vitro studies on the glycemic regulatory effect of food-derived constituents with in vitro DPP-IV inhibitory activity.

Number	Source	Substrate	Cell Line	Benefits	Disadvantage	Reference
1	Egg white	- Fractionation hydrolysate - Peptide: PFL, RVASMASEKM	STC-1	Peptides but not free amino acids, showed a potent GLP-1 and CCK secretagogue effect	Proteins only had a modest GLP-1 secretagogue effect, no CCK release from proteins	[33]
2	Casein	- Whey hydrolysate - GPVRGPFPIIV	GLUTag	Could stimulate GLP-1 release		[153]
3	Bovine hemoglobin	VVYPW, VVYPWQRF, and YPWQRF	STC-1	Potent inhibitors of the GLP-1 inactivating DPP-IV	Did not show significant modulatory effects on the corresponding prohormones mRNA levels	[165]
4	Skin secretions of the crowned bullfrog	Family of tigerinin peptides	GLUTag and BRIN-BD11	- Released GLP-1 from GLUTag -Released Insulin from BRIN-BD11		[167]
5	Frog skin	Hymenochirin-1b (Hym-1B; IKLSPETKDNLKKV- LKGAIKGAIAVAKMV.NH 2)	GLUTag and BRIN-BD11	- Stimulated GLP-1 secretion from GLUTag cells - Increase in the rate of insulin release from BRIN-BD11 cells		[168]
6	Bacterial peptide	(MAADIISTIGDLVK WIIDTVNKFKK), (MAQDIISTIGDLVK WIIDTVNKFTKK), (MAADIISTIGDLVK WIIDTVNKFTKK), and (MAQDIISTIGDLVK WIIDTVNKFKK)	NCI H716 cells			[162]
7	Bovine hemoglobin	ANVST, TKAVEH, and KAAVT	STC-1	- Secretion of CCK and GLP-1 levels - Expression of DPP-IV inhibition action		[164]

**Table A5:** In vivo studies on the glycemic regulatory effect of food-derived constituents with in vitro DPP-IV inhibitory activity.

Food Component	Animal Model	Method Research	Main Findings	Reference
Extract of H. rosa-sinensis leaves	Male Long-Evans rats, streptozotocin (STZ)—induced diabetic	Dose: 250 and 500 mg/kg; oral gavage Duration: 28 days	- Decreased serum glucose, cholesterol, and triglycerides - Increased circulating insulin, hdl cholesterol, and hepatic glycogen without increasing body weight	[50]
Rhazya stricta root extracts	Male BALB/c albino mice, Alloxan-induced diabetic	Dose: 20 mg/kg b.wt/day; oral Duration: 28 days	- Inhibiting enzymes DPP-IV - Increase in the GLP1 secretion - Reduction in blood glucose	[176]
Terminalia arjuna extract	Male Wistar rats, streptozotocin (STZ)—induced diabetic	Dose: 500 mg/kg, oral Duration: 4 weeks	- Superior DPP-IV inhibitory activity	[51]
The extract of Pueraria tuberosa	Male Charles Foster rats, streptozotocin (STZ)—induced diabetic	Dose: a dose of 500 mg/kg bw Duration: 35 days	- Increase in secretion of GLP-1 and GIP - Decrease in DPP-IV specific activities	[178]
Amaranth grain	Male Wistar rats, streptozotocin (STZ)—induced diabetic	Dose: 30 g/day, oral Duration: 12 weeks	- Reduced DPP-IV activity	[179]
Urchin gonad tissue extracts	Male rats, streptozotocin (STZ)—induced diabetic	Dose: 1.8 mg/kg/day, intragastric Duration: 10 days	- Decreased fasting serum glucose levels - Reduced blood malondiadehyde	[56]
Grape seed procyanidin extract	Female Zucker rats	Dose: 25–35 mg/kg/day, oral Duration: 19–60 days	- Decreased DPP-IV activity	[180]
Porcine skin gelatin hydrolysate (<1kDa fraction) released from Flavourzyme	Sprague Dawley male rats, streptozotocin (STZ)—induced diabetic	Dose: 300 mg/day; oral Duration: 42 days	- Improvement of glucose tolerance - Inhibition of DPP-IV activity - Increase in insulin levels - Increase in active GLP-1 levels - Reduction of glucagon levels	[181]
Atlantic salmon skin gelatin hydrolysate released from Flavourzyme	Sprague Dawley male rats, streptozotocin (STZ)—induced diabetic	Dose: 300 mg/day; oral administration Duration: 5 weeks	- Improvement of glucose tolerance - Increased active GLP-1 concentrations - Reduction of glucagon levels - Enhancement of insulin secretion - Inhibition in plasma DPP-IV activity - Improvement of glycemic control	[182]
Rice endosperm and rice bran protein hydrolysate released from pepsin or papain	Male Sprague Dawley rats	Dose: 2 g/kg body weight once (oral); and 500 mg once (ileal)	- No significant difference in plasma glucose during OGTT - Reduced glycemic response - Increased GLP-1 concentrations - Reduced plasma DPP-IV activity - Increased active GLP-1	[183]
Halibut (HSGH) and tilapia (TSGH) fish skin gelatin hydrolysates (<1.5 kDa fraction) released from Flavourzyme	Sprague Dawley male rats, streptozotocin (STZ)—induced diabetic	Dose: 750 mg/kg bw/day; oral Duration: 30 days	- Decreased plasma glucose - Reduced plasma DPP-IV activity - Increased total and active GLP-1 concentrations - Increased plasma insulin levels - TSGH is more potent as an antihyperglycemic agent than HSGH	[55]
Corn zein hydrolysate released from papain	Male sprague Dawley rats	Dose: 500 mg/rat; Ileal, once Duration: acute	- Enhancement of insulin secretion - Increase in total GLP-1 concentration - Decrease in plasma DPP-IV activity - Prevention of hyperglycemia	[184]
Sodium caseinate hydrolysate (<1 kDa fraction) released from bromelain	Sprague Dawley male streptozotocin (STZ)—induced diabetic rats	Dose: 250 and 500 mg/kg/day; administration Duration: 6 weeks	- Improvement of glucose tolerance - Inhibition of plasma DPP-IV activity - Increase of active GLP-1 levels - Enhancement of insulin secretion	[185]
ß-casein-derived peptide LPQNIPPL from gouda-type cheese	Female Sprague Dawley rats	Dose: 300 mg/kg, once, oral administration Duration: acute	- Decreased plasma glucose - No change in insulin plasma concentrations	[187]
Peptide AGFAGĐAPR from Chinese black tea	Male ICR mice, streptozotocin (STZ)—induced diabetic	Dose: 400 mg/day; oral administration Duration: 57 days	- Increase of GLP-1 levels - Increase of insulin levels	[188]
